# MCRWR: a new method to measure the similarity of documents based on semantic network

**DOI:** 10.1186/s12859-022-04578-1

**Published:** 2022-02-01

**Authors:** Xianwei Pan, Peng Huang, Shan Li, Lei Cui

**Affiliations:** School of Health Management, China Medical University, No. 77 Puhe Road, Shenyang North New Area, Shenyang, Liaoning Province People’s Republic of China

**Keywords:** Semantic similarity network, Network analysis, Medical subject headings, Random walk with restart algorithm

## Abstract

**Background:**

Besides Boolean retrieval with medical subject headings (MeSH), PubMed provides users with an alternative way called “Related Articles” to access and collect relevant documents based on semantic similarity. To explore the functionality more efficiently and more accurately, we proposed an improved algorithm by measuring the semantic similarity of PubMed citations based on the MeSH-concept network model.

**Results:**

Three article similarity networks are obtained using MeSH-concept random walk with restart (MCRWR), MeSH random walk with restart (MRWR) and PubMed related article (PMRA) respectively. The area under receiver operating characteristic (ROC) curve of MCRWR, MRWR and PMRA is 0.93, 0.90, and 0.67 respectively. Precisions of MCRWR and MRWR under various similarity thresholds are higher than that of PMRA. Mean value of P5 of MCRWR is 0.742, which is much higher than those of MRWR (0.692) and PMRA (0.223). In the article semantic similarity network of “Genes & Function of organ & Disease” based on MCRWR algorithm, four topics are identified according to golden standards.

**Conclusion:**

MeSH-concept random walk with restart algorithm has better performance in constructing article semantic similarity network, which can reveal the implicitly semantic association between documents. The efficiency and accuracy of retrieving semantic-related documents have been improved a lot.

## Background

PubMed database is the largest biomedical database containing more than 32 million citations and abstracts in 2021. It is updated daily with 200–4000 citations and has an exponentially growing tendency [1]. Besides Boolean retrieval with a query, PubMed provides users with an alternative way called “Related Articles” in the result page, which can recommend relevant documents based on semantic similarity. How to recommend and retrieve relevant documents effectively and accurately from millions of scientific articles remains a very difficult and challenging task. Therefore, it has great importance to explore a more accurate approach to measure the similarity between documents based on semantic relations.

There is no doubt that the ideal method to compute the similarity of documents is based on the full-text analysis [2]. However, considering the cost and limitation of obtaining the full text, it may be more sensible to select the metadata of literature records to measure the similarity of documents, such as MeSH terms, titles, abstracts and references.

Small explored and established relatedness between articles using co-citation analysis [3]. Co-citation occurs if two articles are co-cited by a common article. The more frequently two articles are co-cited, the higher the co-citation intensity is and the higher the similarity of the corresponding documents is. However, since this approach ignored the content information of articles which were fundamental nature of articles, the precision was often not high.

Considering the content information of articles, researchers have proposed various methods to calculate similarity based on semantic relations between documents. Chandrasekaran et al. [4] reviewed and traced the evolution of semantic similarity methods proposed over the years, categorizing them as knowledge-based, corpus-based, deep neural network-based methods, and hybrid methods. Among them, there are five popular approaches to measure the similarity between documents based on titles and abstracts: cosine similarity using term frequency-inverse document frequency (TF-IDF cosine), latent semantic analysis (LSA), topic modeling, and two Poisson-based methods best match 25 (BM25) and PMRA [5–11]. Researchers had shown that PMRA had the best performance among the five methods above. PMRA was developed by integrating TF-IDF and document length of articles into BM25 algorithm, which founded the “related articles” feature of PubMed.

However, PMRA does not always recommend the relevant documents for the topic that users are interested in. For example, if two articles have similar word distributions, they would be inferred as relevant, disregarding the semantic differences between them. And vice versa, PMRA may make wrong recommendations if two articles are highly relevant semantically while having different word distributions. Therefore, to explore a more precise method, we should not only take the word distribution into consideration, but also utilize the implicitly semantic relations between a pair of terms extracted from articles. As two most popular biomedical terminology thesauruses, MeSH thesaurus and unified medical language system (UMLS) metathesaurus are used to explore the semantic relationship between terms [12, 13]. Castro and Berlanga studied the literature similarity using semantic annotations from UMLS, demonstrating that the semanticconcepts were useful to optimize the similarity measurement of documents [2]. Cui and Pan calculated the semantic similarities of documents using the semantic relations between MeSH terms and tried to construct a semantic similarity network of documents in further study [14, 15]. Nevertheless, some studies held different opinions that MeSH terms were not competitive, possibly because they failed to consider the implicit information between MeSH terms.

In order to measure the semantic similarity between documents in a specific domain more accurately, we propose a novel method using random walk with restart (RWR) algorithm to walk the MeSH-concept similarity network which is constructed utilizing the semantic relations of MeSH terms and semantic concepts. RWR algorithm [16–18] is usually used to predict the similarity and relatedness between nodes in a network by choosing a seed node and transiting randomly from the present node to neighboring nodes based on the probabilities between two nodes, and finally getting a convergent probability. In the present study, MeSH terms and semantic concepts are extracted from the downloaded articles and used to represent the documents. According to the similarity of MeSH terms and the co-occurrence frequency between MeSH terms and semantic concepts, we construct a MeSH-concept similarity network and calculate the similarity between documents by RWR algorithm. The proposed method is compared with the PMRA algorithm by the area under ROC curve (AUC), P5 value, precision and document clustering for evaluation.

## Methods

We proposed a new method called MeSH-concept Random Walk with Restart (MCRWR) to measure the semantic similarity of documents in Medline database. There are three steps. Firstly, MeSH terms were extracted from articles and MeSH similarity network was constructed based on the hierarchical relationship of MeSH tree numbers which corresponded to the MeSH terms in the MeSH thesaurus. Secondly, semantic concepts were extracted from titles and abstracts of articles and identified from UMLS (Unified Medical Language System) metathesaurus. MeSH-concept similarity network was constructed by incorporating the semantic concepts into MeSH similarity network. Finally, Semantic similarity between two articles was computed according to the feature vectors generated from MeSH-concept similarity network by Random Walk with Restart (RWR) algorithm. We downloaded 4240 articles from 2005 TREC (Text Retrieval Conference) genome project as the corpus. The performance of our proposed approach was evaluated by four measures (area under ROC curve, precision, P5 value, and document clustering) compared with MeSH RWR(MRWR) algorithm and PMRA (PubMed Related Article) algorithm.

In this study, we use the following four steps.

1. Define and collect a corpus of documents.
2. Extract and pre-process the MeSH terms and semantic concepts from titles and abstracts of documents to generate term-document matrices.
3. Calculate pairwise document-document similarities using PMRA algorithm and RWR algorithm respectively.
4. Evaluate the proposed algorithm (MCRWR) by the area under ROC curve, P5 value, precision and the effect of document clustering.

### Study corpus

The corpus of this study comes from 2005 text retrieval conference (TREC) Genome Project [19] (http://skynet.ohsu.edu/trec-gen/data/2005/genomics.qrels.large.txt), which consists of a subset of MEDLINE from 1994 to 2003 and includes 34,633 articles. In 2005, five generic topic templates (GTTs) including semantic types were created by TREC to reflect the biologists’ information needs. Each topic template has 10 topics. Domain experts judge the relevance between each topic and document being included in the topic, and then assign a corresponding discriminant score for the documents: 0 represents “not relevant”, 1 represents “possibly relevant”, and 2 represents “definitely relevant”. In our study, if the relevance score of topic-document is not equal to 0, we regard the document as one of documents belonging to the topic. Given that two documents have cross-topics, we regard the two documents as relevant, which is defined as golden standards for evaluating the proposed MCRWR method. Among the total 34,633 articles, 4491 articles have topic-relevance score greater than 0. Removing invalid PubMed ID (PMID), 4240 non-overlapping articles on 49 topics (87 articles belonging to 2 topics and 2 articles belonging to 3 topics) are included for the following study. The detail information of the corpus is shown in Table 1.

**Table 1:** The detail information of the studied corpus

ID	Semantic template	Topic code	Related articles
1	Method or protocol	100–109	922
2	Gene(s) & disease	110–119	1413
3	Gene & biological process	120–129	927
4	Genes & function of organ & disease	130–134 136–139	210
5	Gene with mutation & biological impact	140–149	859

### Term-document matrices

In this study, PMID is used as the unique identifier. MeSH terms, as well as titles and abstracts are extracted from the corpus to generate term-document matrixes. Compared with the subheadings and non-major MeSH terms, the major MeSH terms are more specific and can represent the thematic content of articles to a greater degree. Therefore, only major MeSH (hereafter referred to as MeSH) extracted from the documents by MeSH thesaurus (2016 edition) are retained and no further redundant operations are performed.

In calculating the similarity between documents, the core components of PMRA are the term frequencies that co-occur in two documents and the importance of co-occurred terms in the respective document, while the RWR algorithm is based on the MeSH terms and semantic concepts from titles and abstracts being used to build similarity networks. Considering the differences in the selection of textual features between PMRA and RWR algorithms, different methods are adopted to deal with titles and abstracts. We delete the punctuation marks, numbers and redundant blanks in the titles and abstracts for PMRA, then use the Porter algorithm [20] to extract the stem and generate the stem sets. Term frequency and inverse document frequency of every stem from each article are counted. For RWR algorithm, we use Garcia’s approach [2] which adopts the concept mapping annotator (CMA) method [21] to identify semantic concepts in the UMLS metathesaurus (2016AB Edition) to match the semantic concepts in the titles and abstracts. CMA provides candidate concepts and corresponding matching scores in annotating semantic information which is similar to MetaMap [22]. The threshold of matched scores is controlled to a small range to obtain as many as possible semantic concepts. Each identified concept is labeled by the Concept Unique Identifier (CUI) in the UMLS metathesaurus and TF-IDF of each CUI is counted. To build the MeSH-concept network model in the following study more easily, we filter the semantic concepts in each document. Because articles in Medline usually have 3–5 major MeSH terms, we can choose 3–5 concepts for the MeSH-concept network for balance. In this study, we only retain top 5 concepts based on TF-IDF. Semantic concepts identified by CMA would be either a single word (i.e., Gene) or a phrase with specific meaning (i.e., Gene Expression), so the matched concepts contain more abundant semantic information.

Three term-document matrices are built after text pre-processing: MeSH term-document matrix, stem-document matrix and semantic concept-document matrix. The MeSH term-document matrix is a 0–1 matrix, in which the row represents the distribution of MeSH terms in each document and the column represents the MeSH terms corresponding to a particular document. The other two matrices are similar to the MeSH term-document matrix, except that the elements in the two matrices are TF-IDF scores of concepts which have been calculated ahead. To be consistent with the PMRA algorithm, we set the word frequency coefficients of stem and semantic concepts in the titles to 2 and those in the abstracts to 1. The stem-document matrix is used for PMRA algorithm, and the other two term-document matrices are used for RWR algorithm.

### Similarity of documents

#### Similarity of documents based on PMRA

The PMRA algorithm is developed on the basis of BM25, both two algorithms assume that the word frequency of documents theoretically obeyed the Poisson distribution. In measuring document similarity, PMRA first picks out the terms that co-occurred in the titles, abstracts and MeSH terms of two documents, and then calculates the weight of each term in its corresponding document (Eq. 1), finally computes the similarity between two documents based on term weights (Eq. 2). For computational simplicity, only the stemmed terms from titles and abstracts are included in this study.

1 $$W_{t,d} = \frac{{\sqrt {idf_{t} } }}{{1 + \left( {\frac{\mu }{\lambda }} \right)^{k - 1} e^{ - (\mu - \lambda )l} }}$$

In Eq. 1, idf_t_ is the inverse document frequency of the term t in document d. λandμare set according to the default parameters (λ = 0. 022, μ = 0.013) [10], representing whether the term t is the expected probability of the topic which document d belongs to. k is the term frequency of term t, and l is the length of document d.

2 $$Sim.PMRA(d,c) = \sum\limits_{t = 1}^{N} {W_{t,d} *W_{t.c} }$$

In Eq. 2, N is the number of terms that co-occurred in the titles and abstracts of document d and document c. All document similarity scores are normalized in [0,1].

#### Similarity of documents based on RWR

**Fig. 1:**
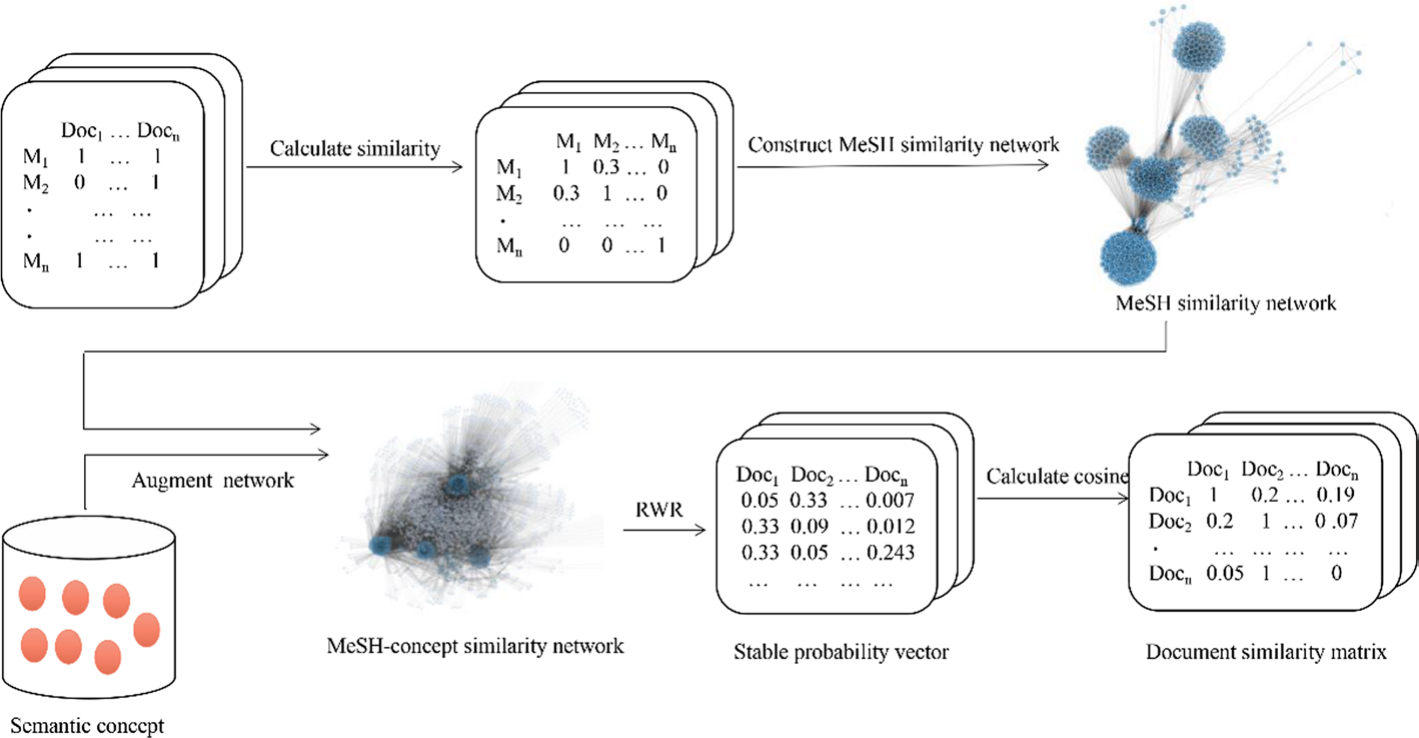
Flowchart of network modelling

Network modeling is performed to calculate the similarity between documents, and the flowchart of the procedure is presented in Fig. 1. Doc_1−n_ means documents in the corpus, M means MeSH terms extracted from the document. Every document was represented by some MeSH terms to construct the MeSH term-document matrix as showed in the upper left in Fig. 1. We calculate the similarity between the MeSH terms based on the frequency and the hierarchical relationship of MeSH tree numbers which corresponded to the MeSH terms in the MeSH thesaurus to obtain MeSH network. After that, semantic concepts are extracted from titles and abstracts of articles and identified from UMLS (Unified Medical Language System) metathesaurus. The MeSH-concept similarity network is constructed by adding semantic concepts to the MeSH similarity network as nodes. In the two similarity networks mentioned above, the nodes denote MeSH terms or semantic concepts. RWR is performed on the MeSH-concept similarity network, taking the corresponding MeSH terms and semantic concepts of each document as seed nodes to simulate random walk on the whole network. Finally, transfer probabilities of each document are obtained based on all nodes in the network. Thus, the similarity between documents is computed by computing cosine value of transfer probability vectors. Finally, document similarity network is constructed as presented in the lower right of Fig. 1.

### MeSH similarity network

Jiang and Conrath hold the point that the similarity between two terms depended on the difference between individual informativeness and common informativeness [23]. The MeSH tree structure which is a directed acyclic graph has the following properties: (i) one MeSH tree number (a node) responds to a MeSH term; (ii) lower MeSH tree number is more specific than the upper ones. In other words, the meaning of the MeSH tree number is more specific from parent node to child node, the lower the MeSH tree number is located, the more informativeness it has. The amount of information on the root node in the MeSH tree is approximately 0. This method takes into account the hierarchical relationship between the MeSH terms, which can be used to calculate the semantic association of the MeSH terms.

According to Zhu et al. [24], in MeSH thesaurus, each node corresponds to a MeSH tree number. We denote the set of all descendants of node v by des(v). For node v1 and node v2, we use cca(v1, v2) to represent the closest common ancestor. For example, v1 = C04.588.945.418.365 and v2 = C04.588.945.418.948.585, then des(v1) is the set of all descendants of node C04.588.945.418.365(including node v1), cca(v1, v2) = C04.588.945.418. Count(v) is the frequency of node v in the corpus, and N is the total number of MeSH terms appearing in the corpus. The informativeness of MeSH tree number v is calculated by Eq. 3. The similarity score between MeSH tree number v1 and v2 is computed by Eq. 4 and Eq. 5. In this study,εis set to 3 [24].

For the reality that each MeSH term may have one or more tree numbers, we use the average maximum match (AMM) [25] to calculate the similarity score between two MeSH terms. Assuming that the tree number set of MeSH term M1 is denoted as m and that of MeSH term M2 is denoted as n, the similarity of two MeSH terms is measured by formula 6. That is, the similarity score between M1 and M2 is the sum of the maximum similarity between any tree number of M1 and all the tree numbers of M2 plus the sum of the maximum similarity between any tree number of M2 and all the tree numbers of M1, which is divided by the sum of the tree numbers of M1 and M2 (|m| and |n| represent the number of MeSH tree numbers).

3 $$I\left( v \right) = - {\text{log}}\left( {\mathop \sum \limits_{vi \in des\left( v \right)} count\left( {vi} \right)/N} \right)$$

4 $$Dist\left( {v_{1} ,v_{2} } \right) = I\left( {v_{1} } \right) + I\left( {v_{2} } \right) - 2*I\left( {cca\left( {v_{1} ,v_{2} } \right)} \right)$$

5 $$Sim\left( {v_{1} ,v_{2} } \right) = e^{{\frac{{ - Dist\left( {v_{1} ,v_{2} } \right)}}{\varepsilon }}}$$

6 $$Sim\left( {M_{1} ,M_{2} } \right) = \frac{{\mathop \sum \nolimits_{{V_{1} \in m}} max_{{V_{2} \in n}} Sim\left( {v_{1} ,v_{2} } \right) + \mathop \sum \nolimits_{{V_{2} \in n}} max_{{V_{1} \in m}} Sim\left( {v_{2} ,v_{1} } \right)}}{\left| m \right| + \left| n \right|}$$

### MeSH-concept similarity network

We refer to the MeSH similarity matrix as an adjacency matrix, and create an undirected weighted network G (V, E), of which the node V_i_ ∈ V represents the MeSH term, the edge E_i,j_ ∈ E represents the relatedness of the MeSH term V_i_ and V_j_, and the weight of edge is the similarity score of V_i_ and V_j_. For a better performance of network modeling, we add nodes and edges to expand the original MeSH similarity network to a MeSH-concept similarity network. Taking five semantic concepts C_i_ = {C_i1_,C_i2_,C_i3_,C_i4_,C_i5_} filtered from document d as example, they are added into the network G(V = V ∪ C_i_) as new nodes, and new edges are added between the five semantic concepts and MeSH terms included in document d, the weights of new edges are TF-IDF (normalized in the range of [0,1]) of the five semantic concepts respectively. Because some of MeSH terms in a document may be the same with the identified semantic concepts, there may exist the case that one MeSH term may link to two or more same semantic concepts. Given the case above, only the edge with maximum weight between MeSH term and semantic concept is reserved. In the processed MeSH-concept similatity network, weights of edges between MeSH term nodes are the similarity of two MeSH terms, and the weights of edges between MeSH term nodes and semantic concept nodes are TF-IDF of the corresponding semantic concepts. Assuming that there are L semantic concepts in the corpus that are different from each other, and the MeSH term nodes are connected with an average of k semantic concepts, then the expanded MeSH-concept similarity network would have | V |+ L nodes and E + k * | V | edges.

### Document similarity network

The principle of the RWR algorithm is to walk randomly along the edges of the network from the seed nodes in the network. For a node or a set of nodes, it is randomly transited to the neighboring nodes or directly returned to the starting node based on certain probabilities. Studies have shown that the transition probability of each node would reach a stable state after performing the iteration of random walk, the probability distribution of the network would not change any more even though it continues to walk [18]. In this statement, the corresponding stable transition probability of each node or set of nodes in the network can be regarded as the similarity score between the current node and the seed node. The sketch of RWR algorithm can be defined as follows:

7 $$p^{(t + 1)} = \left( {1 - \alpha } \right)Wp^{t} + \alpha q$$

where the column vector p^t^ represents the probability distribution of the t-th step in the network, and α represents the probability of returning directly to the seed node, which is called the restart probability. The smaller the value of αis, the wider the range of nodes walk. W is the probabilistic transfer matrix, and W_i,j_ is denoted as the probability transiting from node i to node j. Parameter q is the restart column vector, representing the probability of each node being a start node(seed node) in the initial statement. In the process of random walk, the iteration is performed by Eq. 7 until p converges and finally a stable probability distribution between the seed node and other nodes is obtained.

In the present study, we use a dRWR function from R package dnet to calculate the RWR process. Based on a term-document matrix, which is a 0–1 matrix suggesting whether the MeSH terms and concepts occurring in a document, we apply dRWR on the MeSH-concept network, by using MeSH terms and semantic concepts in each document as a set of seed nodes for the random walk. Finally, the convergent transition probability distribution of each set of seed nodes are obtained, which can be regarded as the textual feature vector of the corresponding document. In this network, the weighted adjacency matrix is the probabilistic transfer matrix W, the restart vector q of nodes corresponding to MeSH terms and semantic concepts are set to 1, the others are set to 0. In order to prevent the seed node walk too far in the network, α is set to 0.6 after carrying out many experiments to investigate the actual performance of the algorithm. In the iteration process, when the difference between P^t+1^ and p^t^ is less than or equal to 10^−10^, the recognized p is convergent, and obtain the probability distribution of seed nodes.

If we denote MeSH-concept network as the text feature space of the whole corpus, the convergent transfer probability vector of document d could be regarded as the relatedness value of other nodes in the network relative to document d. Thus, we can recognize the convergent transfer probability vector as feature vector of document d. RWR is performed to get the feature vector p_d1_ and p_d2_ of document d1 and document d2 respectively, then similarity between two documents is calculated by cosine similarity function (Eq. 8).

8 $$Sim.RWR\left( {d_{1} ,d_{2} } \right) = \cos \left( {pd_{1} ,pd_{2} } \right) = \frac{{pd_{1} ,pd_{2} }}{{\left| {pd_{1} } \right|*\left| {pd_{2} } \right|}}$$

The article similarity network based on MeSH-concept RWR is constructed, and the same process is done on the MeSH similarity network to create the article similarity network based on MeSH RWR.

### Evaluation indicators

To demonstrate the practical significance of using semantic concepts to enhance the MeSH similarity network, we perform the RWR algorithm on the MeSH-concept similarity network and the MeSH similarity network. For comparing the performance of PMRA with those of two RWR algorithms, taking the golden standard of TREC as reference, we measure the area under curve (AUC) at corpus level, the precision of different similarity thresholds at topic level, P5 values and the clustering effects of documents based on the topological structure of article similarity networks.

Disregarding the difference of document topics, the documents are divided into two categories according to the TREC golden standard: document pairs within the same topic and document pairs among different topics. Selecting a series of similarity thresholds, we draw the ROC curve using true positive rate (TPR in Eq. 9) as the ordinate and false positive rate (FPR in Eq. 9) as the abscissa. The ROC curve could identify the advantages and disadvantages of algorithms under any similarity threshold. The larger the AUC is, the better the algorithm performs.

**Fig. 2:**
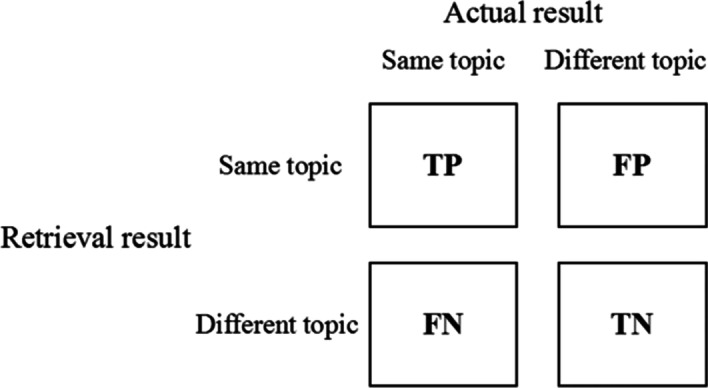
Definitions of TP, FP, FN, TN

Biologists tend to be keen on quality rather than quantity in retrieving literature related to the topic of interest. PubMed, for example, recommends only the top five "Related Articles" articles. Meanwhile, considering that the similarity between different topics might be different, we rank the documents for every topic based on similarity scores, and compute the precision of top five documents ranked (P5 in Eq. 10). The P5 value of each topic is the average P5 values of all documents in the topic. The P5 value of the corpus is the mean P5 values of all topics. In addition, we selecte several similarity thresholds, regarding document-document pair as unsimilar if the similarity between them is lower than the threshold, and computed the precision of each topic and the whole corpus (Precision in Eq. 10). The definitions of true positive (TP), false positive (FP), true negative (TN) and false negative (FN) are shown in confusion matrix in Fig. 2. We use Chi-square test to compare the performance of different algorithms.

Because the TREC corpus do not provide direct similarity scores, it classifies the documents according to the topic. Therefore, we cluster the documents based on the topological structure of the document similarity network compared with the golden standard. To get a better performance of document clustering, we utilize the Infomap [26] algorithm from the Igraph package [27] in R language [28], which is considered to be one of popular network community detecting algorithms at present. Infomap algorithm regards the coding length of random walk path in graph as the objective function to be optimized and transformed the community detection in the network into the issues of information compressing and coding [29].

9 $$TPR = \frac{TP}{{TP + FN}},FPR = \frac{FP}{{FP + TN}}$$

10 $$P5 = \frac{TP}{N}\left( {N = 5} \right),\;Precision = \frac{TP}{{TP + FP}}$$

## Results

### MeSH-concept similarity network

**Fig. 3:**
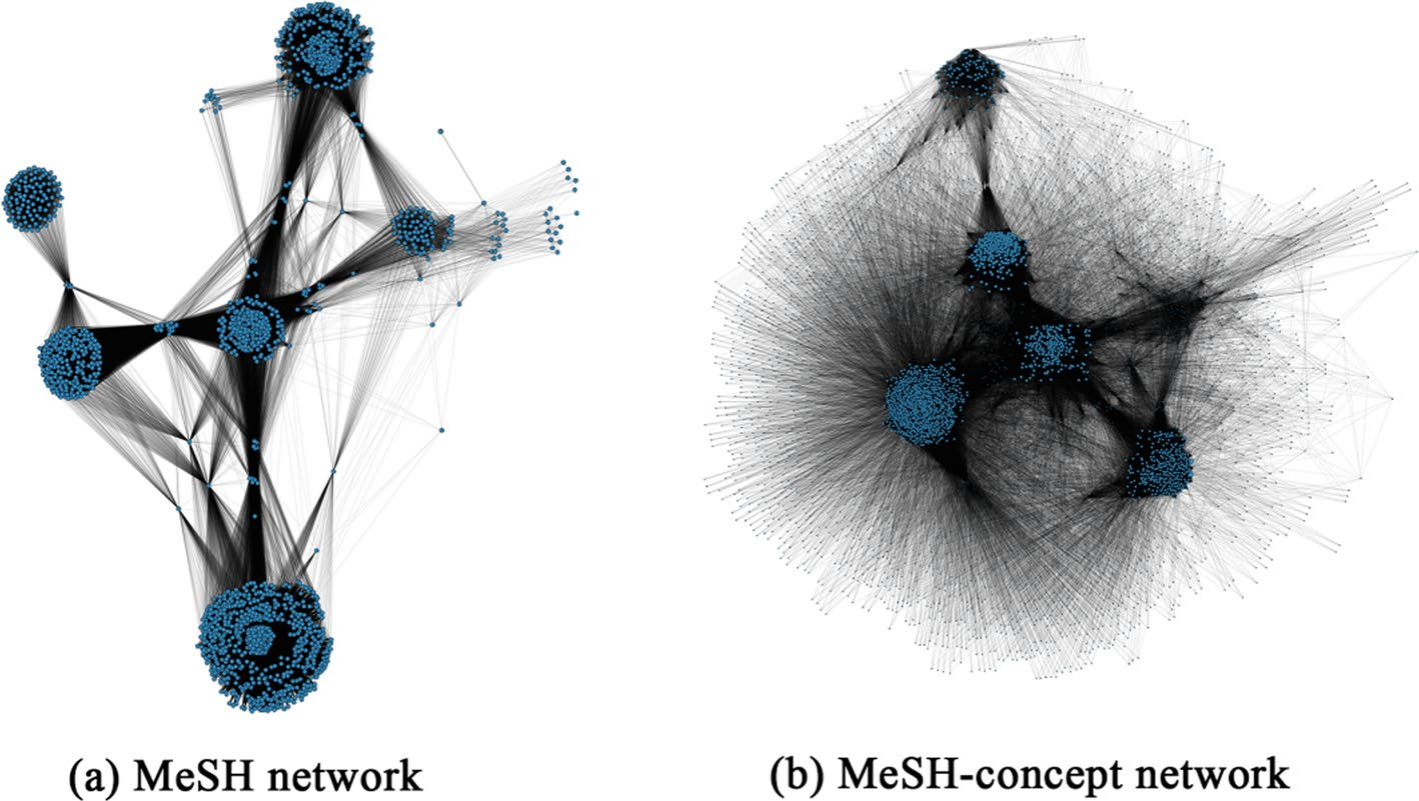
Visualization of MeSH network and MeSH-concept network

Basic information about the MeSH similarity network and the enhanced MeSH-concept similarity network are shown in Table 2. An important attribute of network is clustering coefficient, which is an indicator to reveal the clustering degree of nodes in the whole network. The bigger the clustering coefficient is, the closer the nodes are linked with others. Both networks have distinct community structures and are closely connected between nodes (Fig. 3). In contrast, the scale of theMeSH-concept similarity network is larger, but the degree of clustering decreases with a lower clustering coefficient. The semantic concept nodes in MeSH-concept similarity network surround the MeSH term nodes to form a radial shape.

**Table 2:** Basic information of MeSH network and MeSH-concept network

Net. type	No. of nodes	No. of MeSH nodes	No. of concept nodes	No. of edges	Average degree	Clustering coefficient
MeSH net	2438	2438	0	366,614	300.75	0.66
MeSH-concept net	7302	2438	4864	417,538	114.36	0.58

### ROC curve, P5, and precision

**Fig. 4:**
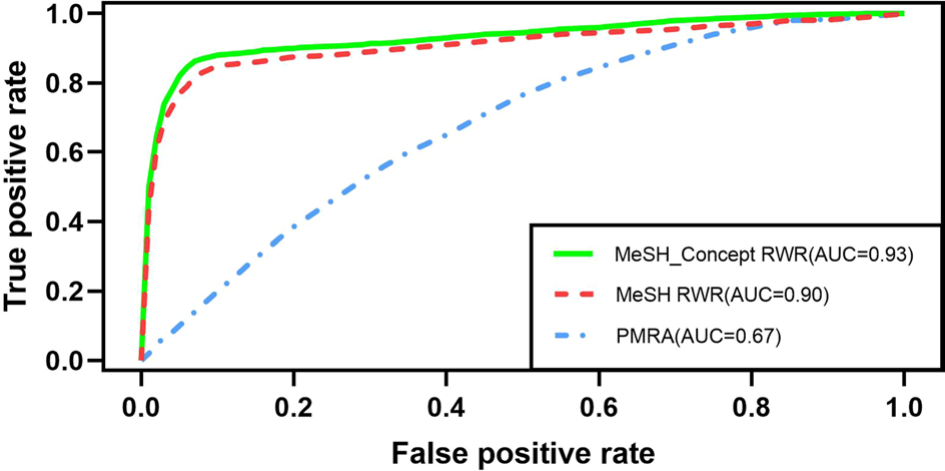
ROC curves of MCRWR, MRWR and PMRA algorithms

According to the TREC golden criterion, the document-document pairs in the document similarity matrix are divided into two categories: the doc-doc pairs within the same topic and the doc-doc pairs between different topics. By Wilcoxon rank-sum test, there is a significant difference in similarity scores between the two categories of doc-doc pairs using all three algorithms (MCRWR, MRWR, PMRA) (P < 2.2E-16), which indicates that the three algorithms could identify whether the documents belong to the same topic to a certain extent. The ROC curves of the three algorithms are shown in Fig. 4. MCRWR algorithm have the largest AUC (0.93, 95% confidence interval (CI): 0.892–0.961), MRWR algorithm is slightly inferior to MCRWR algorithm with AUC = 0.90(95% CI: 0.874–0.932), while the AUC of PMRA algorithm is the smallest (0.67, 95% CI: 0.653–0.71).

**Fig. 5:**
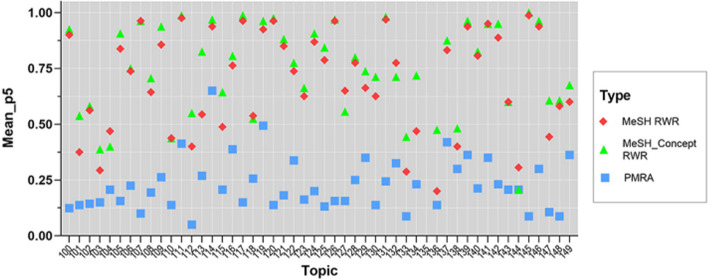
Mean P5 values of 49 topics

The mean P5 values of 49 topics are shown in Fig. 5. Mean P5 values are reported with the format mean ± standard deviation. Mean P5 values based on MCRWR (0.742 ± 0.204) are obviously higher than those of MRWR (0.692 ± 0.226) and PMRA (0.223 ± 0.119). For the topics (104, 133, 136 et al.) containing less than 5 documents, the mean P5 values of all three algorithms are less than 0.5. The mean P5 values of PMRA algorithm are lower than those of the two RWR algorithms on all topics except topic 44 holding equal mean P5 values to MCRWR. In the two RWR algorithms, the method based on the MeSH-concept similarity network performs better in 44 topics. The comparisons of mean P5 values of all three algorithms are statistically significant with P < 0.001. However, the results of P5 values could only reveal the precision of the top 5 related documents, so there exist some drawbacks that we can’t observe the precision of all related documents.

**Fig. 6:**
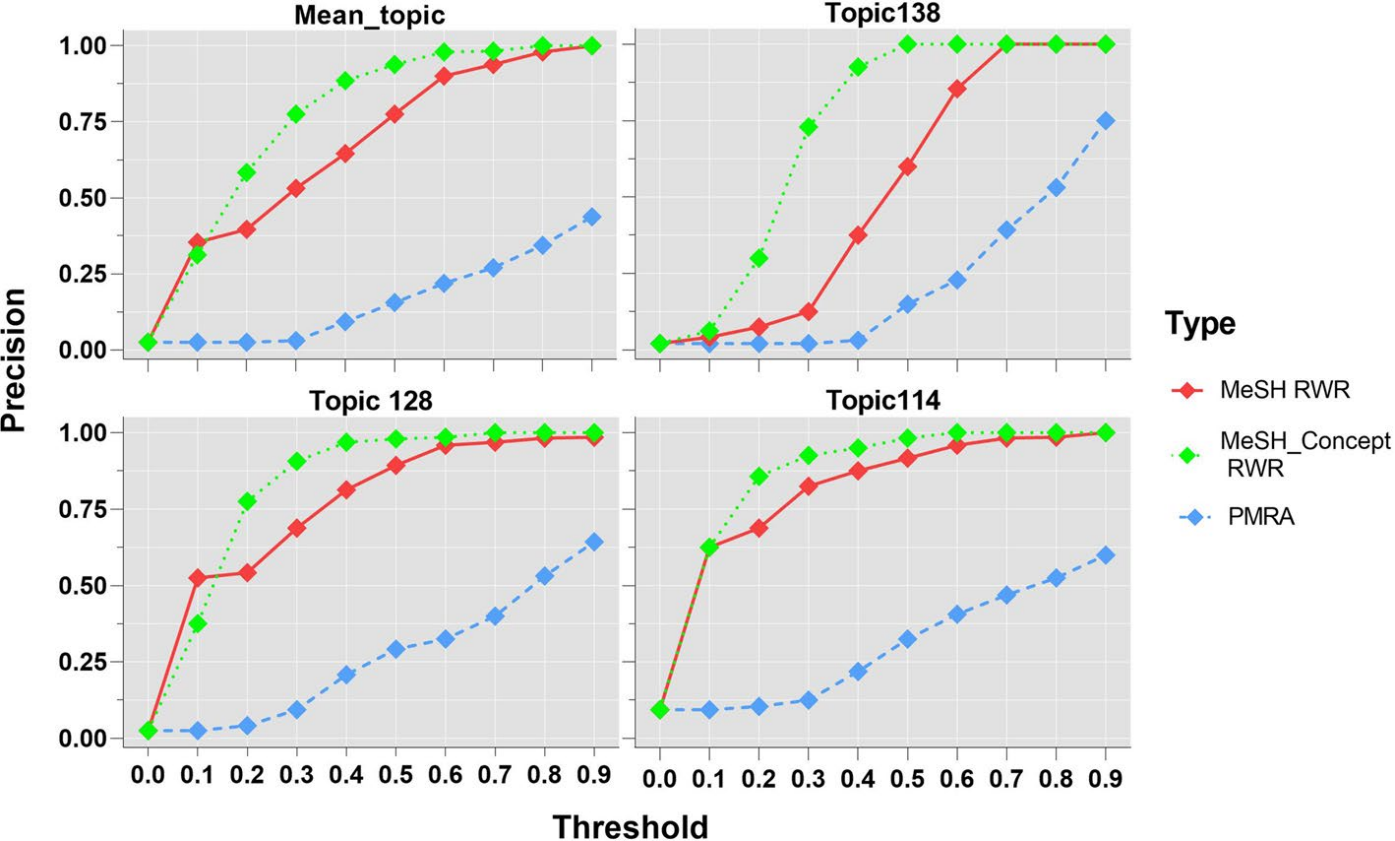
Precision curves of topics for three algorithms

In order to improve the persuasiveness of the results, we calculate the precision of every topic under the similarity thresholds of 0 to 0.9 after comparing the areas under ROC curve and mean P5 values. As shown in Fig. 6, the upper-left graph illustrates the average precision rate for all topics, the other three subgraphs present the precision rates for the three randomly chosen topics (138, 128, 14) respectively. The three curves at the initial points (threshold = 0) in the graph are nearly the same, and increase with the growth of similarity threshold. The precision curves of the two RWR algorithms are above that of PMRA under all similarity thresholds. The precision curve of MeSH-concept RWR rises rapidly under the similarity thresholds of 0 to 0.5, and then approaches to 1 with little change afterwards. The precision of MeSH RWR is higher than that of MeSH-concept RWR when the similarity thresholds are less than or equal to 0.1, and the precision curve increases steeply to the peak in the similarity thresholds of 0.2 to 0.7. PMRA precision curve rises faster with the increase of similarity thresholds, which indicates that PMRA algorithm is sensitive to high similarity documents. The comparisons of precisions of three algorithms are statistically significant with P < 0.05(P = 0.002 of Mean_topic, P = 0.03 of Topic138, P = 0.003 of Topic128, and P = 0.001 of Topic114).

### Network clustering

**Fig. 7:**
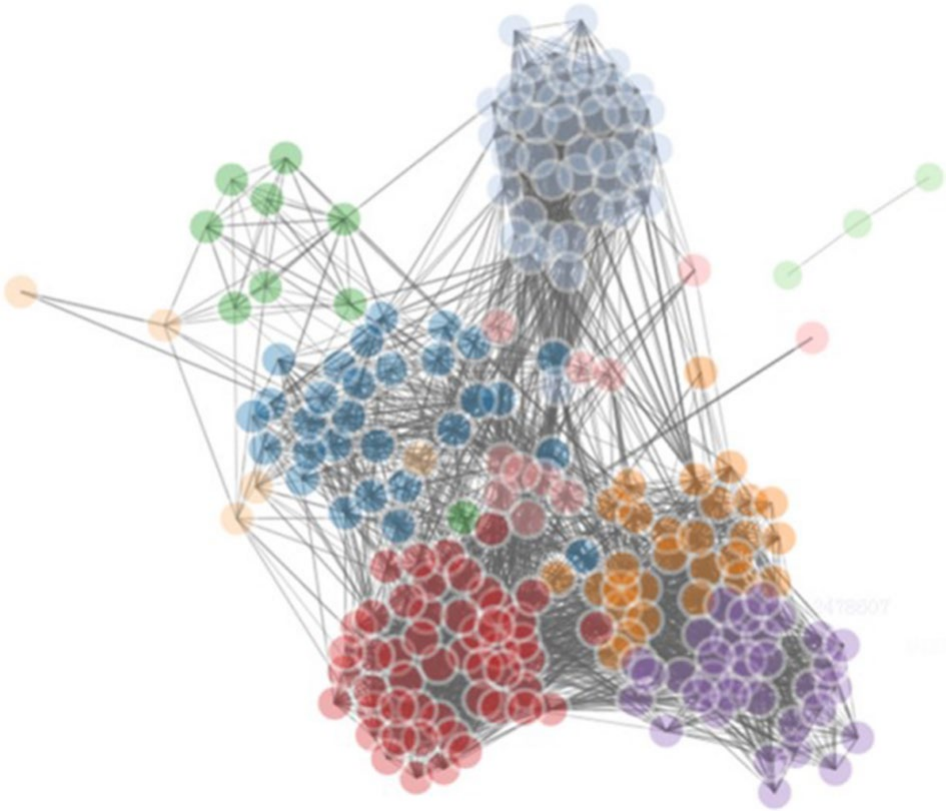
The pruned document similarity network of “Genes&Function of organ & Disease”

Considering a simple attempt of testing and suitable number of articles, We take 210 articles from 9 topics in the fourth semantic template "Genes&Function of organ & Disease" as nodes, and construct an undirected weighted similarity network based on MeSH-concept RWR, of which the edge weights are the similarity scores between documents. When the document similarity network is constructed, the topological structure of the network is very indecipherable and it is tough to explain and read for us. To more clearly elucidate the performance of document clustering, we need to prune the network using a similarity threshold. Given a similarity threshold, if the similarity score between two nodes is lower than the threshold, the line between the two nodes will be deleted, if the similarity score between two nodes is bigger than the threshold, the line between the two nodes will be retained. After testing a lot of similarity thresholds, we find that the similarity threshold of 0.08 is a cut-off value to make the network more explainable. With different datasets, the similarity thresholds are different. It is necessary to perform many tests to find a suitable similarity threshold. Being pruned using the similarity threshold of 0.08, the processed network (see Fig. 7) contain 210 nodes and 4119 edges, the same color nodes represent the same topic according to the golden standard. A total of eight communities are detected after network clustering. The related document sets of four communities (2, 3, 7, 8) are identical to those of four topics classified by the golden standard. Community 4 is a subset of topic 132, and community 1 contain all the documents from topic137 and one document from topic 132. Community 5 include all the documents of topic 130 and two papers from topic 133. Community 6 contain all the documents on topic 134 and three articles of topic 133. Specific information on clustering results and golden standard are presented in Tables 3 and 4 respectively.

**Table 3:** Topics and corresponding document numbers of golden standards

Topic ID	130	131	132	133	134	136	137	138	139
Article number	29	42	28	5	9	3	50	11	33

**Table 4:** Communities and corresponding article numbers of the pruned network clustering

Cluster ID	1	2	3	4	5	6	7	8
Article number	51	42	33	27	31	12	11	3

## Discussion

**Fig. 8:**
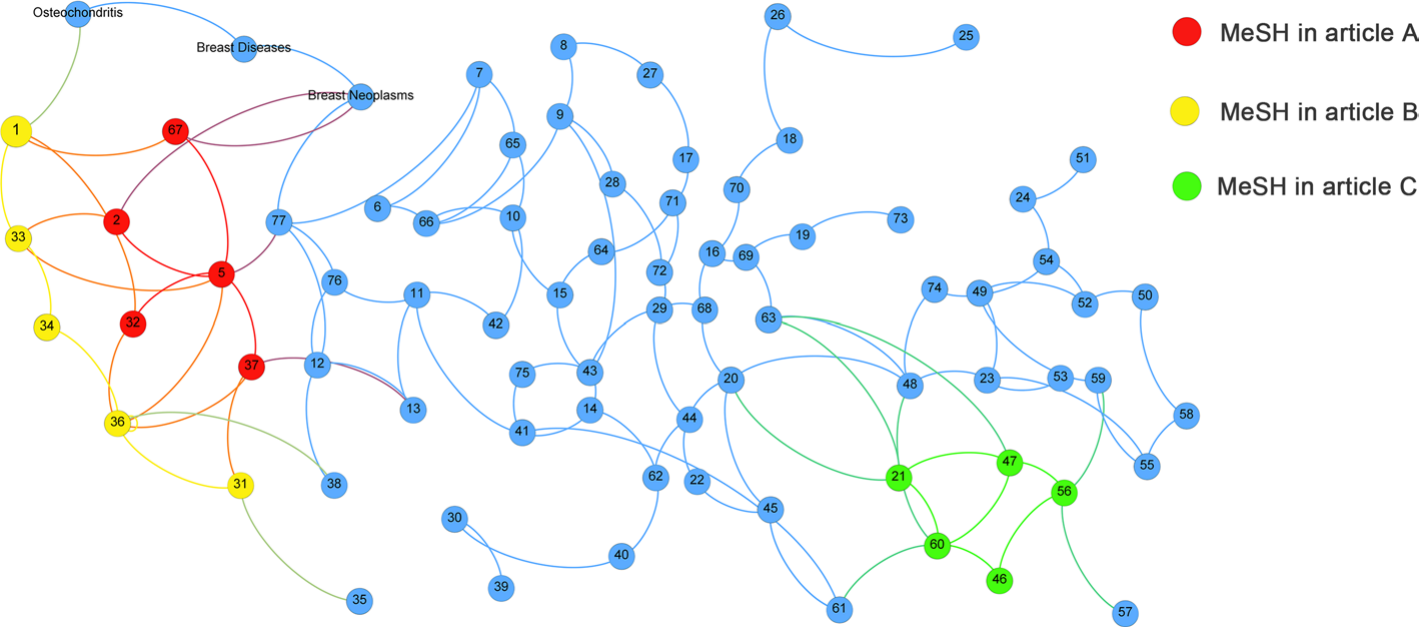
Example of document similarity network based on MeSH terms

Several methods were investigated in [30] to calculate the similarity between MeSH terms, and measures to compute the similarity of documents based on the similarity of MeSH terms were presented. However, this approach only considered the direct similarity between MeSH terms, and ignored the indirect relationship of mutual transition between them, which might undiscover the hidden semantic information between documents. The RWR algorithm used on the MeSH similarity network could solve this tough problem of the MeSH similarity transition to a great degree. Figure 8 showed a simple MeSH similarity network. There was no link between "Osteochondritis" and "Breast Neoplasms", but both were neighboring nodes of "Breast Diseases". If we arbitrarily considered them having no relationship, we would arrive to ignore the bridging role via the bridging term of "Breast Diseases". In the RWR method, when random walker transited from the seed node "Osteochondritis" to other nodes, "Osteochondritis" would jump to "Breast Neoplasms" by the bridging term "Breast Diseases". In addition, the more closely the seed MeSH terms of the two articles were linked in the network, the higher the similarity between the two documents was. In Fig. 8 for example, the similarity between article A represented by the red nodes and article B represented by the yellow nodes was obviously higher than the similarity between article A and article C represented by the green nodes.

**Fig. 9:**
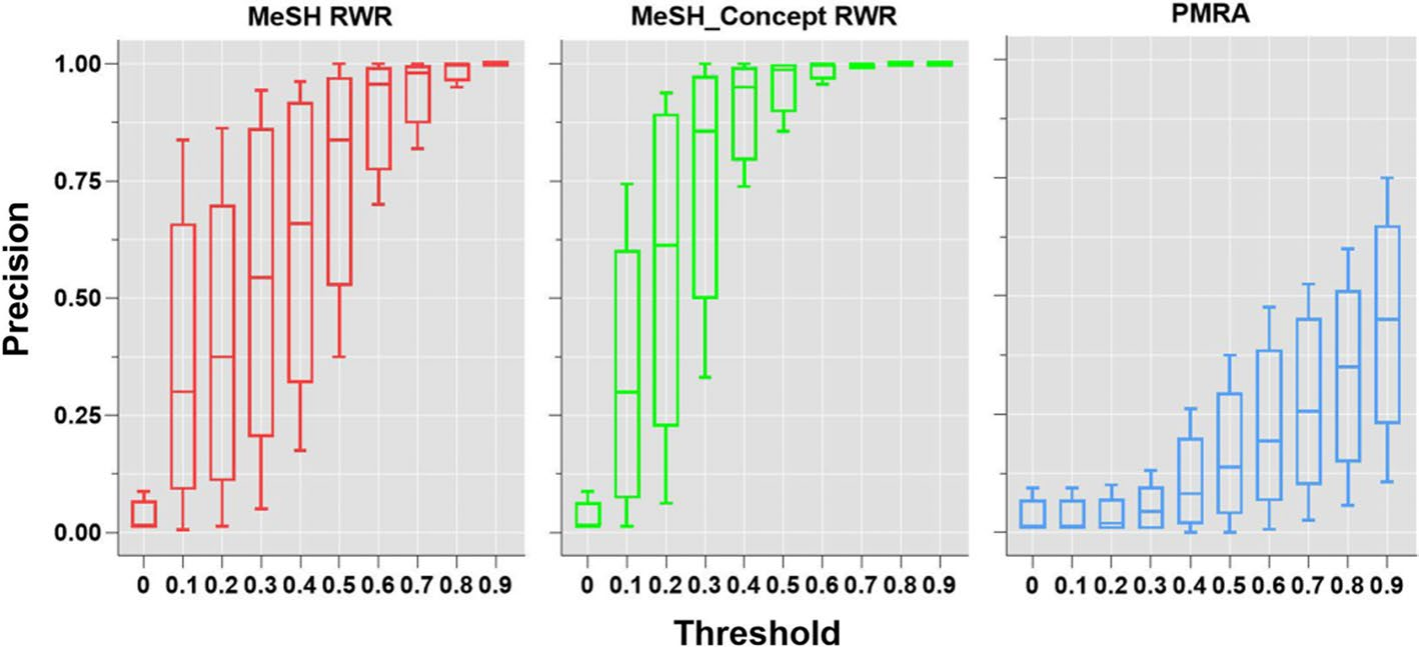
Boxplots of topic precisions at different thresholds by three algorithms

Because of the difference in precision between different topics, there might be a bias that the precisions of document similarities were represented only by mean precision of topics which was macro-averaged. Therefore, we draw boxplots of topic precisions under different thresholds (Fig. 9). Obviously, in addition to the mean precision, the quartile of topic precision of the RWR algorithm was also higher than that of the PMRA algorithm at the same similarity threshold. Precision differences among different topics varied with thresholds, which might be due to differences in the number of documents and the actual content of 49 topics in the corpus.

The proposed method to measure document similarity can not only retrieve the "Related Article" of a certain document, but also perform unsupervised clustering on document collection according to the semantic information. Specific communities can also be detected according to the topological structure of the document similarity network. Meanwhile, we can provide biologists with information navigations through interconnections of document nodes in the network. Since our method is based on the semantic information of documents, we can provide strong supporting evidences for evaluating the importance of documents by identifying the important nodes (such as nodes having high degree of centrality) in the document network.

In this study, MeSH terms and semantic concepts identified from titles and abstracts were used as feature vectors of documents to measure document similarity, thus the calculated document similarity varied dynamically with different corpuses, which was consistent to the reality. With the birth of new knowledge and new literature, the similarity between documents is by no means inflexible. It is well known that there are only coarse-grained hierarchical relationships between nodes in the MeSH classification system. This superficial annotation of semantic relationships greatly limited the performance of our algorithm. When adding edges between nodes of semantic concepts and those of MeSH terms, we simply took TF-IDF as the edge weights and did not consider the semantic relationship between MeSH terms and semantic concepts. To compensate for these shortcomings, we will utilize 134 semantic types of UMLS semantic network and 54 kinds of semantic relationships to combine MeSH terms with semantic concepts [13] in the future work. By using fine-grained second-level mapping of semantic types and semantic relationships, we can analyze document similarities in greater depth.

## Conclusion

We propose a new approach to measure document similarities based on the network semantic space by combining semantic information with the network model. In constructing a network model, it is better to combine semantic concepts with MeSH terms than to use MeSH terms only. MeSH-concept RWR is superior to PMRA of PubMed by comparing AUC, P5 values, precision and network clustering, which suggests that MeSH-concept RWR has great application prospects in classifying and clustering documents. Moreover, it also provides a new perspective for evaluating documents.

## Data Availability

The datasets used and analysed during the current study are available from the corresponding author on reasonable request.

## Abbreviations

MeSH: Medical subject headings
MCRWR: MeSH-concept random walk with restart
MRWR: MeSH random walk with restart
PMRA: PubMed related article
ROC: Receiver operating characteristic
RWR: Random walk with restart
TF-IDF: Term frequency-inverse document frequency
LSA: Latent semantic analysis
UMLS: Unified medical language system
TREC: Text retrieval conference
GTTs: Generic topic templates
PMID: PubMed ID
CMA: Concept mapping annotator
CUI: Concept unique identifier
BM25: Best match 25
AUC: Area under the curve
TPR: True positive rate
AMM: Average maximum match
FPR: False positive rate
TP: True positive
FP: False positive
TN: True negative
FN: False negative

## References

[1] PubMed Overview. https://pubmed.ncbi.nlm.nih.gov/about/. Accessed 20 Mar 2021.

[2] Garcia Castro LJ, Berlanga R, Garcia A (2015). In the pursuit of a semantic similarity metric based on UMLS annotations for articles in PubMed Central Open Access. J Biomed Inform.

[3] Small H (1973). Co-citation in the scientific literature: a new measure of the relationship between two documents. J Am Soc Inform Sci.

[4] Chandrasekaran D, Mago V (2021). Evolution of semantic similarity—a survey. ACM Comput Surv.

[5] Boyack KW, Newman D, Duhon RJ, Klavans R, Patek M, Biberstine JR, Schijvenaars B, Skupin A, Ma N, Börner K (2011). Clustering more than two million biomedical publications: comparing the accuracies of nine text-based similarity approaches. PLoS ONE.

[6] Salton G, Buckley C (1988). Term-weighting approaches in automatic text retrieval. Inf Process Manag.

[7] Deerwester S, Dumais ST, Furnas GW, Landauer TK, Harshman R (1990). Indexing by latent semantic analysis. J Am Soc Inf Sci.

[8] Blei DM, Ng AY, Jordan MI (2003). Latent Dirichlet allocation. J Mach Learn.

[9] Sparck Jones K, Walker S, Robertson SE (2000). A probabilistic model of information retrieval: development and comparative experiments Part 1. Inf Process Manag.

[10] Sparck Jones K, Walker S, Robertson SE (2000). A probabilistic model of information retrieval: development and comparative experiments Part 2. Inf Process Manag.

[11] Lin J, Wilbur WJ (2007). PubMed related articles: a probabilistic topic-based model for content similarity. BMC Bioinf.

[12] Rogers F (1963). Medical subject headings. Bull Med Libr Assoc.

[13] Bodenreider O. The Unified Medical Language System (UMLS): integrating biomedical terminology. Nucleic Acids Res. 2004;32(Database issue):D267–70. 10.1093/nar/gkh061 PMC308795 14681409

[14] Pan XW, Yang Y, Cui L (2013). Research review of scientific paper network and conception of constructing paper similarity network. J Med Inf.

[15] Pan XW. Comparison and evaluation of content and semantic similarity article network construction methods. China Medical University, 2014.

[16] Suratanee A, Plaimas K (2015). DDA: a novel network-based scoring method to identify disease-disease associations. Bioinform Biol Insights.

[17] Sun J, Shi H, Wang Z, Zhang C, Liu L, Wang L, He W, Hao D, Liu S, Zhou M (2014). Inferring novel lncRNA-disease associations based on a random walk model of a lncRNA functional similarity network. Mol Biosyst.

[18] Lovász L. Random walks on graphs: a survey. Combinatorics. 1996: 353–398.

[19] Hersh WR, Bhupatiraju RTTREC (2005). Genomics track overview. Trec Proc.

[20] Rijsbergen C, Robertson SE, Porter MF. New models in probabilistic information retrieval. 1980.

[21] Berlanga R, Nebot V, Jimenez E (2010). Semantic annotation of biomedical texts through concept retrieval. Procesamiento Del Lenguaje Natural.

[22] MetaMap—a tool for recognizing UMLS concepts in text. https://lhncbc.nlm.nih.gov/ii/tools/MetaMap.html. Accessed 8 Oct 2021.

[23] Jiang JJ, Conrath DW. Semantic similarity based on corpus statistics and lexical taxonomy. Rocling. 1997:11512–0.

[24] Zhu SF, Zeng J, Mamitsuka H (2009). Enhancing MEDLINE document clustering by incorporating MeSH semantic similarity. Bioinformatics.

[25] Wang JZ, Du Z, Payattakool R, Yu PS, Chen CF (2007). A new method to measure the semantic similarity of GO terms. Bioinformatics.

[26] Rosvall M, Bergstrom CT (2008). Maps of random walks on complex networks reveal community structure. Proc Natl Acad Sci USA.

[27] Csardi G, Nepusz T. The igraph software package for complex network research. InterJournal. 2006:1695.

[28] Team R. R: A language and environment for statistical computing. 2013. Computing. 2011;1:12–21.

[29] Rosvall M, Axelsson D, Bergstrom CT (2009). The map equation. Eur Phys J Spec Top.

[30] Zhou J, Shui Y, Peng S, Li X, Mamitsuka H, Zhu S (2015). MeSHSim: an R/Bioconductor package for measuring semantic similarity over MeSH headings and MEDLINE documents. J Bioinform Comput Biol.

